# Pre-transplant IE1-specific T-cell response and CD8^+^ T-cell count as predictive markers of treated HCMV reactivation in kidney transplant recipients

**DOI:** 10.3389/fimmu.2025.1538795

**Published:** 2025-04-16

**Authors:** Federica Zavaglio, Irene Cassanti, Marilena Gregorini, Maria Antonietta Grignano, Teresa Rampino, Daniele Lilleri, Fausto Baldanti

**Affiliations:** ^1^ Microbiology and Virology Department, Fondazione IRCCS Policlinico San Matteo, Pavia, Italy; ^2^ Department of Clinical, Surgical, Diagnostic and Pediatric Sciences, University of Pavia, Pavia, Italy; ^3^ Department of Internal Medicine and Therapeutics, University of Pavia, Pavia, Italy; ^4^ Unit of Nephrology and Dialysis and Transplantation, Fondazione IRCCS Policlinico San Matteo, Pavia, Italy

**Keywords:** pre-transplant immunity, human cytomegalovirus, kidney transplant, immunological markers, T-cell response

## Abstract

**Background:**

Human cytomegalovirus (HCMV) infection represents a significant complication for kidney transplant recipients (KTRs). The goal of this study was to evaluate potential immunological markers at pre-transplant in HCMV-seropositive KTRs for predicting HCMV severe reactivation (e.g treated HCMV reactivation) during the first year after transplant.

**Methods:**

Before transplant, lymphocyte count was measured in whole blood and HCMV-specific T-cell response was determined using ELISpot assay after stimulation with pp65, IE-1 and IE-2 peptides pool. HCMV DNA was monitored during the first year after transplant. Among the 65 KTRs enrolled, 44 (68%) patients had HCMV self-resolving reactivation (Controllers) while 21 (32%) required antiviral treatment for HCMV reactivation (Non-Controllers).

**Results:**

No significant difference in CD4 T-cell count was observed, but Controllers had higher CD8^+^ T-cell counts compared to Non-Controllers. Based on ROC analysis, a CD8^+^ T-cell count ≥215 cells/μl was associated with a lower incidence of HCMV reactivation after transplant. Additionally, a higher IE-1-specific T-cell response was observed in Controllers and patients with IE1-specific T-cell response ≥60 spots showed a reduced incidence of HCMV reactivation and lower DNAemia peak.

**Discussion:**

Lymphocyte counts and HCMV-specific T-cell response can be measured at pre-transplant in KTRs in order to efficiently predict the risk of treated HCMV reactivation during the first year after transplant. Potential cut-off and diagnostics algorithm should be better investigated in a large patients setting.

## 1 Introduction

Human Cytomegalovirus (HCMV) infection still represents one of the most important opportunistic infection in solid organ transplant recipients (SOTRs) (1). Two approaches have been proposed for control of HCMV prevention, pre-emptive therapy (PET), which involves a PCR-guided administration of anti-HCMV treatment to patients at risk for HCMV disease (i.e., monitoring the blood viral load and giving antiviral drugs to patients at predetermined levels of viral load), and universal prophylaxis (i.e., administration of antiviral drugs to all transplanted patients for 6-12 months) (2, 3). Although it is widely known that patients who are HCMV-seronegative at transplant and receive the organ from a HCMV-seropositive donor (D+/R-) are at higher risk of HCMV infection, HCMV-seropositive recipients (R+) may be at risk of reactivation in the post-transplant period, especially in relation to the type of transplanted organ and immunosuppressive therapies (4–6).

To date, the assessment of immunological tools able to predict the spontaneous clearance of HCMV infection in HCMV-seropositive SOTRs represents a crucial milestone for the success of transplant. In this setting, monitoring of lymphocytes subsets in SOTRs could be used as simple approach for stratification of the risk of HCMV infection, reactivation or relapse after treatment (7–12). Moreover, HCMV-specific T cells are crucial for the prevention of HCMV disease, observing that both CD4^+^ and CD8^+^ HCMV-specific T cells are involved in the first line of specific cellular immune response in HCMV-seropositive transplanted recipients, as well as in a long term control of reactivation (13–18). Furthermore, the risk of high-level DNAemia and consequently early treatment is reduced in those patients with higher T-cell response between 2 and 4 week post-transplant (16, 19).

On the other side, the evaluation of pre-transplant HCMV-specific immune response seems to be useful for a preliminary patients’ stratification of the risk of HCMV reactivation in HCMV-seropositive recipients (20–25). However, the role of pre-transplant HCMV-specific T-cell response and its potential use in clinical practice should be better elucidated.

In this study, we aimed to evaluate HCMV-specific T-cell response at pre-transplant in HCMV-seropositive kidney transplant recipients in order to investigate the predictive role in the stratification of HCMV DNAemia and requirement of antiviral treatment.

## 2 Materials and methods

### 2.1 Patients enrolment and HCMV monitoring

HCMV-seropositive kidney transplant recipients were consecutively enrolled at Nephrology and Dialysis Department of IRCCS Policlinico San Matteo in Pavia (Northern Italy). At baseline (day of transplant), heparinized whole blood samples were collected for peripheral blood mononuclear cells (PBMC) isolation and lymphocyte T cell count. Detailed flow-chart representing patients’ enrollment, follow-up and stratification is included in **Supplementary Figure 1** (**Supplementary Figure S1**).

All the patients were treated with induction therapy with anti-thymocyte globulin (ATG; 1 mg/kg/die for three days) or anti-CD25 monoclonal antibody (basiliximab; 20 mg at time of transplant and 20 mg at the fourth day post-transplant). Methylprednisolone was added in both cases. Triple immunosuppressive standard regimens was also administered after transplant (cyclosporin or tacrolimus/micophenolic acid or mycophenolatemofetil/methylprednisolone), according to therapeutic protocols. All the analysis were performed according to our Institutional Review Board and written informed consent was obtained by all enrolled patients (Protocol number 20180004199).

### 2.2 HCMV management and infection definitions

After transplant, HCMV DNAemia was monitored according to diagnostic protocols. In detail, HCMV DNAemia was monitored in whole blood weekly for the first 8 weeks and subsequently every 15 days until the 4^th^ month, then monthly until first year after transplant.

HCMV DNA was quantified using in-house real-time PCR performed on blood samples (26) with some modification. In detail, extraction QIAsymphony^®^ DSP DNA Mini kit (Qiagen; Hilden, Germany) (200 µl of extraction volume) and QuantiFast Pathogen PCR kit (Qiagen) were used for DNA extraction and DNA amplification, respectively.

In case of suspected tissue invasive disease (TID), a tissue biopsy (gastrointestinal disease) or a bronco-alveolar lavage (BAL) fluid sample (pneumonia) was collected for HCMV DNA quantification and histopathological analysis. HCMV disease was defined as possible, probable, or proven according to Ljungman et al. (27).

Self-resolving HCMV DNAemia was defined as the detection of HCMV DNA in blood at any level with subsequent spontaneous clearance without antiviral treatment. Clinically significant HCMV infection was defined as HCMV infection requiring antiviral treatment (either as pre-emptive therapy or for treatment of HCMV disease).

### 2.3 Peripheral blood mononuclear cells isolation

Peripheral blood mononuclear cells (PBMC) were isolated from heparinized whole blood samples by density gradient centrifugation (Lymphoprep, Axis-Shield, Norway) and resuspended in culture medium (RPMI 1640 supplemented with 2mM L-glutamine, 100U/mL penicillin and 100 µg/mL streptomycin and 10% of heat inactivated fetal bovine serum (Euroclone, Italy). Isolated PBMC were stored in nitrogen liquid using freezing medium (65% RPMI 1640 supplemented with 2mM L-glutamine, 100U/mL penicillin and 100 µg/mL streptomycin, 25% human albumin (Grifolds Biologicals, CA, USA) and 10% DMSO (Sigma-Aldrich, MO, USA). Before the use, PBMC were thawed, washed, resuspended in cultured medium and rested overnight at 37°C in a 5% CO_2_ humidified atmosphere (28).

### 2.4 Synthetic peptides

For the evaluation of HCMV-specific T-cell response three peptide pools representative of the whole proteins pp65, IE1 and IE2 were used (JPT Peptide Technologies, Germany). All peptides were 15 aminoacids in length with an overlap of 11 aminoacids, representing a good compromise for stimulation of both CD4^+^ and CD8^+^ T cells (29). Pp65 peptide pool was composed by 138 peptides, IE1 by 120 peptides and IE2 by 143 peptides. Peptides were dissolved in DMSO (Sigma-Aldrich) and diluted in RPMI 1640 medium supplemented with 2mM L-glutamine, 100U/mL penicillin and 100 µg/mL streptomycin. Aliquotes were stored at - 20°C until use. All peptide pools were used at the final concentration of 0,25 µg/mL for each peptide.

### 2.5 HCMV-specific T-cell response detected by ELISpot assay

HCMV-specific T-cell response was determined by ELISpot assay, using ELISpot IFN-γ Basis kits from ELITech (Milan, Italy) according to manufacturer’s instructions.

The Multitestplates (MTP) fitted with membranes and coated with anti-human IFN-γ antibody were supplied in the test kit. PBMC (2x10^5^ cells/100 µl per well) were added in duplicate and stimulated with100 µl of antigen solution or culture medium only (negative control) or phytoheamagglutin (PHA; 5 µg/ml, Sigma-Aldrich). Plates were incubated from 20 to 24 hours at 37°C 5%CO_2_ humidified atmosphere. After cells remove, the alkaline phosphatase (AP)-labeled secondary antibody was added. Two hours later a substrate solution (BCIP/NBT) was added. After several washes under running water, plates were dried. Spots per counted using automated AID ELISpot reader system (AutoImmunDiagnostika GmbH, Germany).

### 2.6 Lymphocyte count

Fresh whole blood was stained with anti-CD3-PC5, anti-CD45-FITC, anti-CD4-RD1 and anti-CD8-ECD monoclonal antibodies (Beckman Coulter, Milan, Italy). After lysis of red blood cells, the absolute number of CD3^+^, CD3^+^CD4^+^ and CD3^+^CD8^+^ T-cell counts were determined by flow cytometry (Navios, Beckman Coulter) using Flow-Count Fluorospheres (Beckman Coulter).

### 2.7 Data analysis

The mean number of spots obtained from duplicate wells was adjusted to 10^6^ PBMC. The mean number of spots/million PBMC obtained by culture medium only was subtracted by the mean number of spots/million PBMC in response to the corresponding antigen in order to obtain the net spots/million PBMC. Results were then given as net spots/million PBMC (later in the text defined as “spots”). Quantitative variables were shown in terms of median or mean values and interquartile range (IQR) while categorical variables were presented as number or percentage. Mann-Whitney test and Fisher’s test were used for data analysis, as well as receiver-operator characteristic (ROC) analysis. Log-rank test was used for the evaluation of cumulative incidence. The best cut-off to predict the spontaneous clearance of HCMV infection at pre-transplant was calculated according to the Youden Index. A multivariate logistic regression was also performed. All the statistical analysis were performed by using GraphPad Prism 8.3.0 (GraphPad Software Inc, CA, USA). All tests were two tailed and *p* value<0.05 was considered significant.

## 3 Results

### 3.1 Patients

Sixty-five HCMV-seropositive KTRs (47 males and 18 females; median age 51 years, [IQR 46-61]) were enrolled at time of transplant. HCMV serological status was positive in 39 (60%) donors, negative in 8 (12.3%) donors and un-known in 18 (27.7%) donors. Clinical characteristics of the patients are shown in **Table 1**. Overall, 44/65 (68%) patients showed at least one self-resolved HCMV reactivation event or undetectable HCMV DNA during the follow up period and were defined as “Controllers”, while 21/65 (32%) were treated for clinically significant HCMV reactivation and were defined as “Non-Controllers”.

**Table 1:** Clinical and demographic patients’ characteristics.

Characteristics	All patients (n=65)	Controllers (n=44)	Non-Controllers (n=21)	*p value*
Age, median [IQR]	51 (46–61)	50 (45–57)	59 (47–64)	**0.046**
Gender, n (%):				
Male	47 (72)	32 (73)	15 (71)	0.999
Female	18 (28)	12 (27)	6 (29)	
Donor Serostatus n (%)				
HCMV positive (D+) HCMV negative (D-) HCMV unknown	39 (60) 8 (12) 18 (28)	27 (62) 8 (18) 9 (20)	12 (57) 0 9 (43)	0.069
Primary Diagnosis, n (%)				
Polycystic kidney	14 (22)	8 (18)	6 (29)	0.352
Nephropathy	13 (20)	11 (25)	1 (5)	0.084
Glomerulonephritis	7 (11)	5 (11)	2 (19)	0.999
Nephroangiosclerosis	5 (7)	3 (7)	2 (9)	0.654
Other	14 (22)	9 (20)	6 (29)	0.535
Unknown	12 (18)	8 (18)	4 (19)	0.999
Induction Therapy, n (%):				
Anti-CD25	51 (78)	35 (79)	16 (76)	0.988
ATG	14 (22)	9 (21)	5 (24)	
Immunosuppressive regimen, n (%):				
Cya, MMF, Steroids	8 (12)	4 (9)	4 (19)	0.420
FK-506, MMF, Steroids	53 (82)	37 (84)	16 (76)	0.502
FK-506, Steroids	1 (2)	0	1 (5)	0.323
Everolimus, FK-506, MMF, Steroids	3 (4)	3 (7)	0	0.545

ATG, anti-human thymocyte globulin; Cya, Cyclosporien A; FK506, tacrolimus; MMF, mycophenolate mofetil. The value in bold refers to a significant difference.

**Figure 1:**
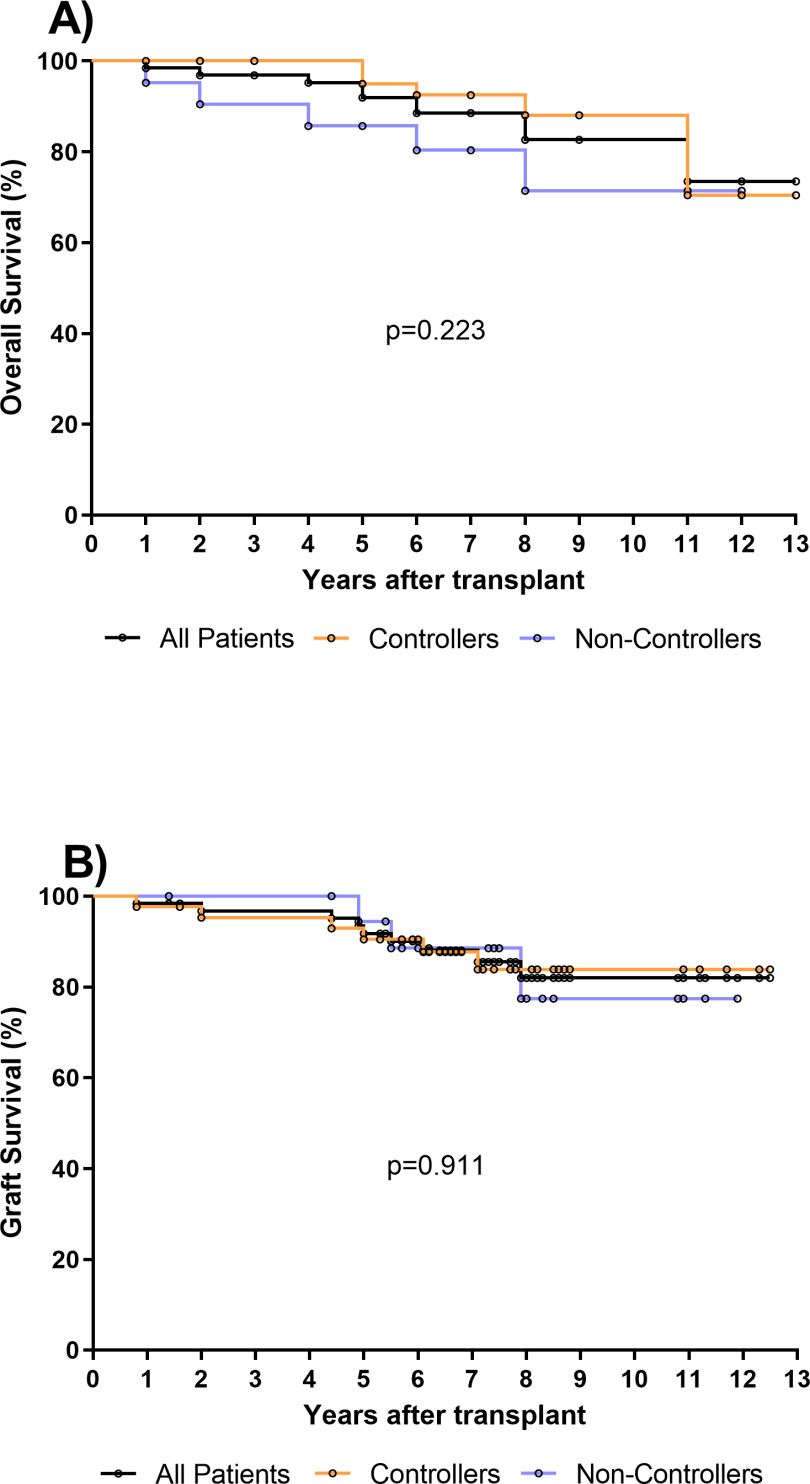
**(A)** Overall survival was evaluated in all kidney transplant recipients (KTR, n=65) (black line), in 44 Controllers (orange line) and in 21 Non-Controllers (purple line). No difference in overall survival was observed between controllers and non-controllers (*p*=0.223). **(B)** Graft survival was evaluated in all kidney transplant recipients (KTR, n=65) (black line), in 44 Controllers (orange line) and in 21 Non-Controllers (purple line). No difference of graft survival was observed between controllers and non-controllers (*p*=0.911).

There were no significant differences in baseline characteristics between the two groups of Controllers and Non-Controllers, except for the age at time of transplant (*p*=0.046). Additionally, even if the difference is not statistically significant, the rate of HCMV seropositive donors was higher in Non-controllers (*p*=0.069) (**Table 1**). The median follow-up after transplantation was 7.2 years (IQR 5.9–8.5 years) for the entire cohort of patients, 7 years (IQR 5.9–8.5 years) for Controllers and 7.3 years (IQR 5.4–8.4 years) for Non-Controllers. Overall, 10/65 (15%) patients died, and 5 of them were Non-Controllers (**Figure 1A**). Based on our results, the overall survival in controllers seems to be higher than that measured in non-controllers. However, this difference is not statistically significant. Regarding the graft survival, 9/65 (14%) patients had graft failure and 3 of them were Non-Controllers (**Figure 1B**).

### 3.2 Pre-transplant absolute number of total CD8^+^ T cell as predictive marker of spontaneously resolving HCMV reactivation during the first year post-transplant

**Figure 2:**
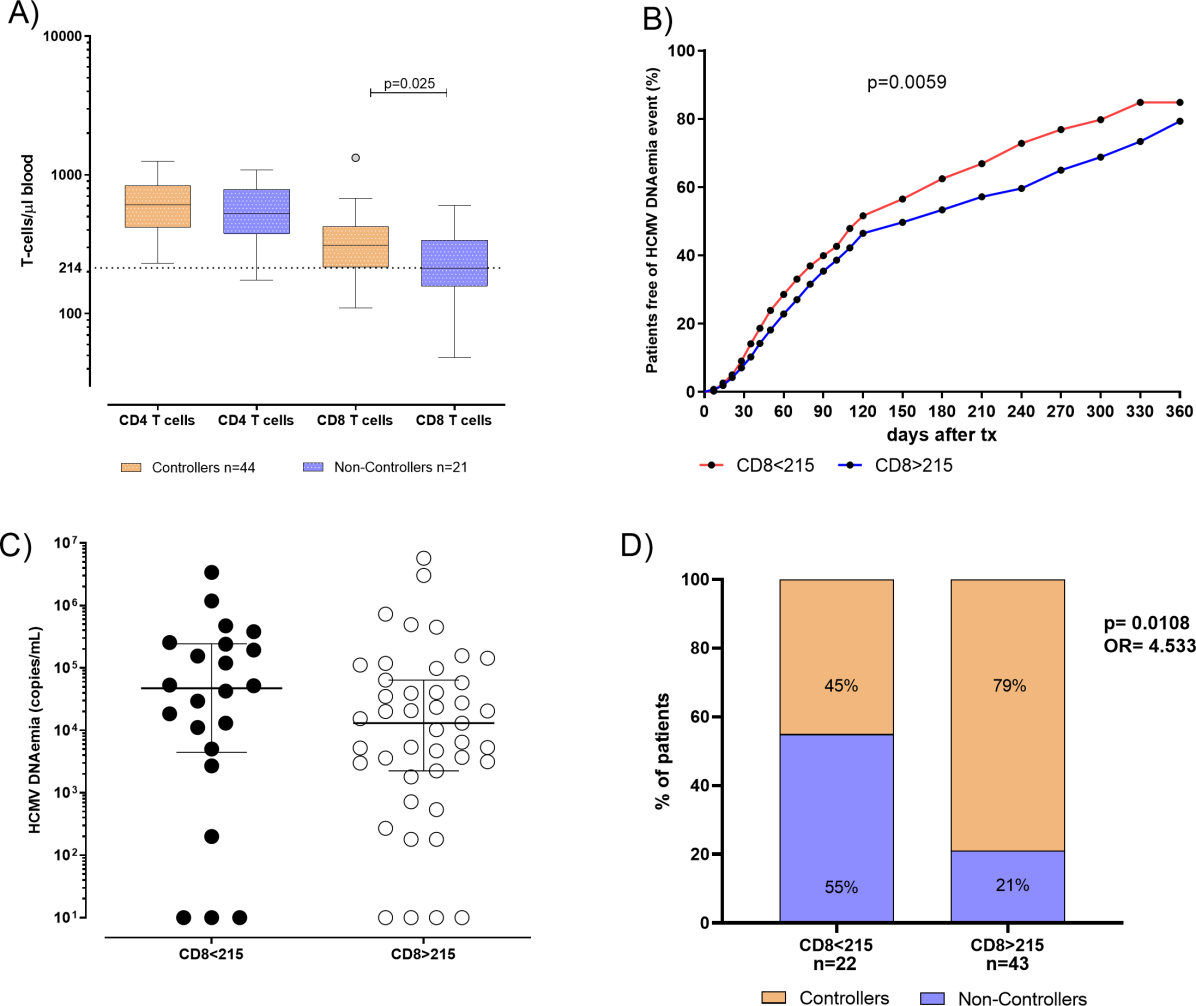
**(A)** Absolute number of total CD4 and CD8 T cells were evaluated and compared in 44 patients Controllers (orange bars) for and 21 Non-Controllers (purple bars). Median of total CD4 and CD8 T cells were shown in the graph as well as significant *p* value. **(B)** Cumulative incidence of HCMV reactivation events in patients absolute number of total CD8 T-cell response<215 CD8 T cells/µl (red line) and in patients with absolute number of total CD8 T-cell response ≥ 215 CD8 T cells/µl (blue line). **(C)** HCMV DNAemia peak in kidney transplant recipients (KTR) was measured in 22 patients with total CD8 T. **(D)** Percentage of Controllers (orange bars) and Non-Controllers patients (purple bars) according to absolute number of total CD8 T-cell response<215 CD8 T cells/µl and in patients with absolute number of CD8 T-cell response ≥ 215 CD8 T cells/µl. P value and Odd ratio (OR) were also given.

The pre-transplant absolute number of total CD4^+^ and CD8^+^ T cells in blood was compared in 44 Controllers and 21 Non-Controllers at pre-transplant. No difference was observed in terms of the median of total CD4^+^ T cell between Controllers and Non-Controllers (610 [IQR 418-838] vs 528 [IQR 377-788] T-cell/μl, respectively) while the median of total CD8^+^T cell was found to be higher in Controllers than Non-Controllers (310 [IQR 215-424] vs 212 [IQR 157-338] T-cell/μl, respectively, *p*=0.025) (**Figure 2A**). In order to predict the spontaneous clearance of HCMV infection based on the absolute number of CD8^+^ T cell, the ROC curve analysis was performed. The optimal cut-off value of 215 CD8^+^ T cell/μl was selected using the Youden index (AUC: 0.67, 95% CI: 0.52-0.82, *p*=0.025) (**Supplementary Table 1**).

Interestingly, the cumulative incidence of HCMV reactivations during the first year after transplant in patients with the absolute number of CD8^+^< 215 T cell/μl was 85%, while in patients with the absolute number of CD8^+^ ≥215 T cell/μl it was 79% (*p*=0.005, **Figure 2B**). HCMV DNAemia at peak was measured and compared in 22 patients with the absolute number of CD8^+^< 215 T cell/μl and in 43 patients with CD8^+^ ≥ 215 T cell/μl in blood. We observed that median of HCMV DNAemia at peak was 47295 [IQR 4455-243863] copies/mL in patients with the absolute number of CD8^+^< 215 T cell/μl in blood, and 13050 (2250–64150) copies/mL in patients with the absolute number of CD8^+^ ≥ 215 T cell/μl in blood (**Figure 2C**), even if this difference was not statistically significant (p=0.169). Regarding patients with the absolute number of CD8^+^< 215 T cell/μl, 12 out of 22 (55%) were Non-Controllers, while 10 out of 22 (45%) patients were Controllers. On the other hand, patients with the absolute number of CD8^+^ ≥ 215 T cell/μl, 9 out of 43 (21%) were Non-Controllers, while 34 out of 43 (79%) were Controllers (*p*=0.018, **Figure 2D**).

### 3.3 Pre-transplant IE1-specific T-cell response as a second predictive marker of spontaneously resolving HCMV reactivation during the first year post-transplant

**Figure 3:**
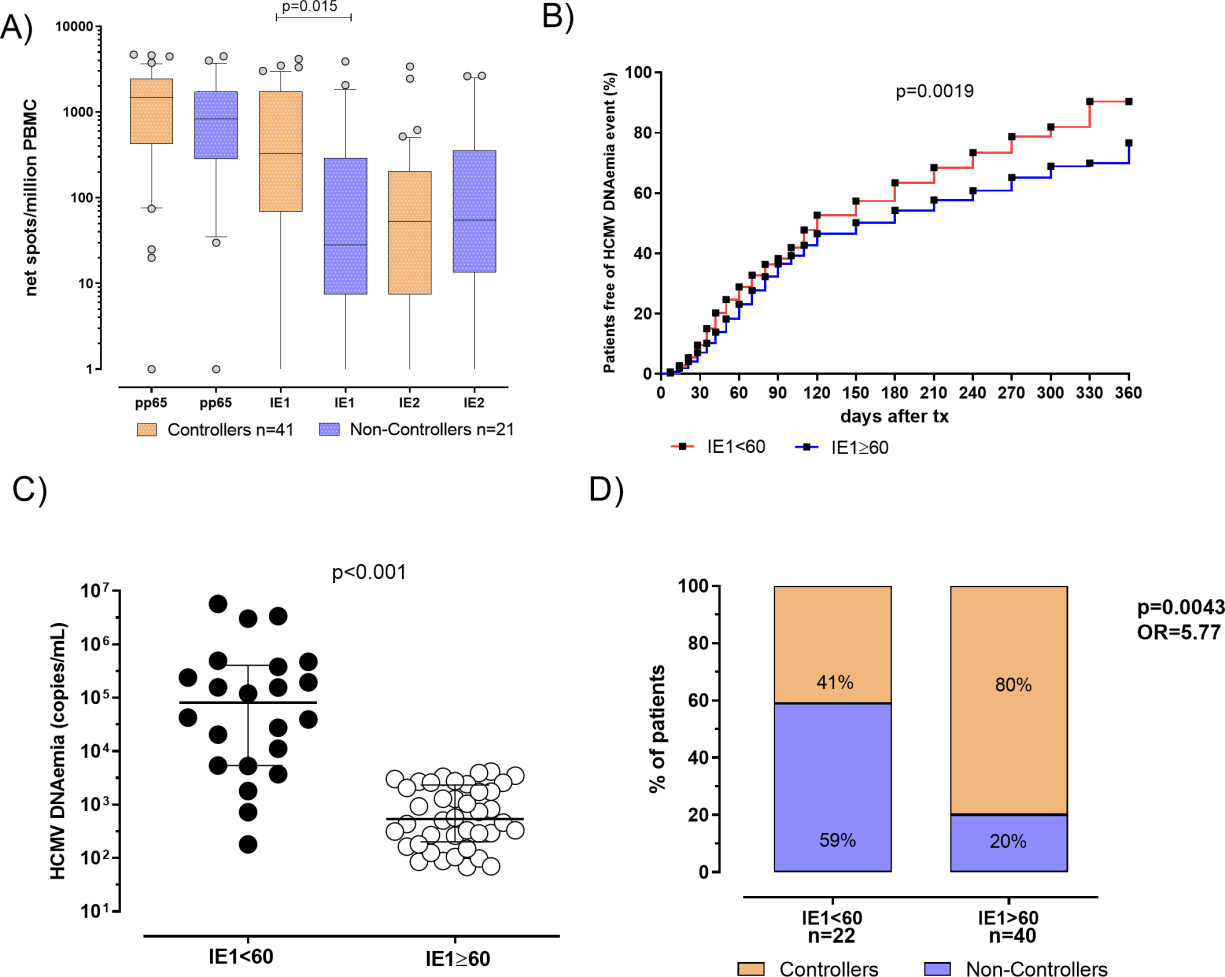
**(A)** Pp65, IE1 and IE2 specific T-cell responses were evaluated and compared in 41 patients Controllers (orange bars) for and 21 Non-Controllers (purple bars). Median antigen-specific T-cell responses were shown in the graph as well as each *p* value. **(B)** Cumulative incidence of HCMV reactivation events in patients with IE1-specific T-cell response<60 spots (red line) patients with IE1-specific T-cell response ≥ 60 spots (blue line). **(C)** HCMV DNAemia peak in patients classified according to pre transplant IE1-specific T-cell response. **(D)** Percentage of Controllers (orange bars) and Non-Controllers patients (purple bars) according to pre-defined IE1-specific T-cell response cut-off of 60 spots. P value and Odd ratio (OR) were also given.

HCMV-specific T-cell response was evaluated in 62 patients (41 Controllers and 21 Non-Controllers). Both pp65 and IE2-specific T-cell response did not significantly differ between Controllers and Non-Controllers (p=0.193 and p=0.869, respectively). On the contrary, a significantly higher median IE1-specific T-cell response was observed in Controllers compared to Non-Controllers (330 [IQR 69-1744] vs 28 [IQR 7-292] spots, respectively; p=0.015) (**Figure 3A**). Additionally, a negative correlation between DNAemia peak and IE1-specific T-cell responses was observed (p=0.0092, r= -0.33 IC 95% between -0.54 and -0.07). Based on these results, a ROC curve analysis was used to predict the spontaneous clearance of HCMV infection and cut-off of 60 spots of IE1-specific T-cell response was calculated using Youden index (**Supplementary Table 1**).

In patients with IE1-specific T-cell response< 60 spots the cumulative incidence of HCMV reactivation events was 90%, while in patients with IE1-specific T-cell response ≥ 60 spots a cumulative incidence of HCMV reactivation events of 77% was observed (p=0.0019, **Figure 3B**). HCMV DNAemia at peak was measured and compared in the two groups of patients, showing that median of HCMV DNAemia at peak was 81325 [IQR 5378-404325] copies/mL in patients with IE1-specific T-cell response< 60 spots and 8390 (337–50085) copies/mL in patients with IE1-specific T-cell response ≥ 60 spots (*p*<0.001, **Figure 3C**). Among patients with an IE1-specific T-cell response at pre-transplant<60 spots, 13 out of 22 (59%) were Non-Controllers, while 9 out of 22 (41%) were Controllers. Otherwise, looking at patients with an IE1-specific T-cell response ≥ 60 spots, 8 out of 40 (20%) were Non-Controllers, while 32 out of 40 (80%) were Controllers (*p*=0.004, **Figure 3D**). In other words, since a higher proportion of Controllers patients showed an IE1-specific T-cell response ≥ 60 spots, measuring HCMV-specific T-cell response at baseline might be used for identifying patients with high rate of self-resolving HCMV reactivation in the post-transplant period.

### 3.4 The use of combined immunological markers might be used for optimizing the HCMV management of transplanted patients

**Figure 4:**
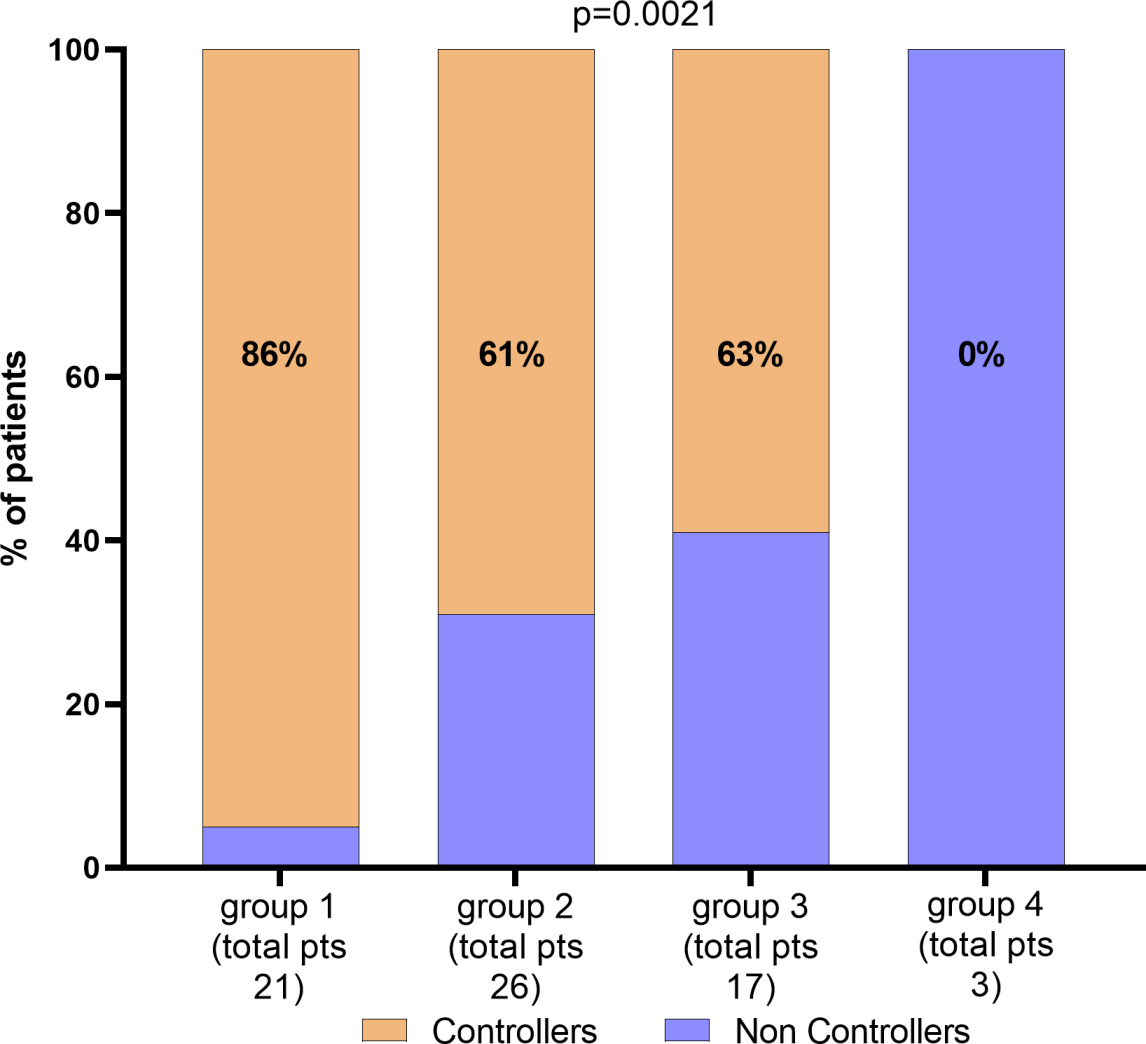
Percentage of patients according with CD8 T-cells, IE1 spots levels and age. Total number of patients and percentage of Controllers have been given for each group. Parameters for the classification of each group have been described in the text.

Based on these findings, age at time of transplant, CD8^+^ T-cell count and IE1-specific T-cell response can be independently used for predicting the risk of treatment for severe HCMV reactivation in HCMV seropositive KTRs. Then, we combined the parameters for identifying the percentage of non-controllers in each group as shown in **Figure 4**. Groups were classified according to age lower than 60 years, high CD8^+^ T cell count (≥215 cells/µl), and high level of IE1-specific T-cell response (≥60 spots). In detail, group 1 included patients with all the three markers (n=21), group 2 included patients at two of the three markers (n=26) while group 3 included patients with only one of the markers described (n=12). Group 4 included patients with none of the markers (n=3). The number and percentage of controllers and non-controllers were given for each group. Interestingly, among group 1, only one of the 21 patients was treated for uncontrolled HCMV infection. On the other hand, all the three patients of group 4 were treated for uncontrolled HCMV infection.

**Figure 5:**
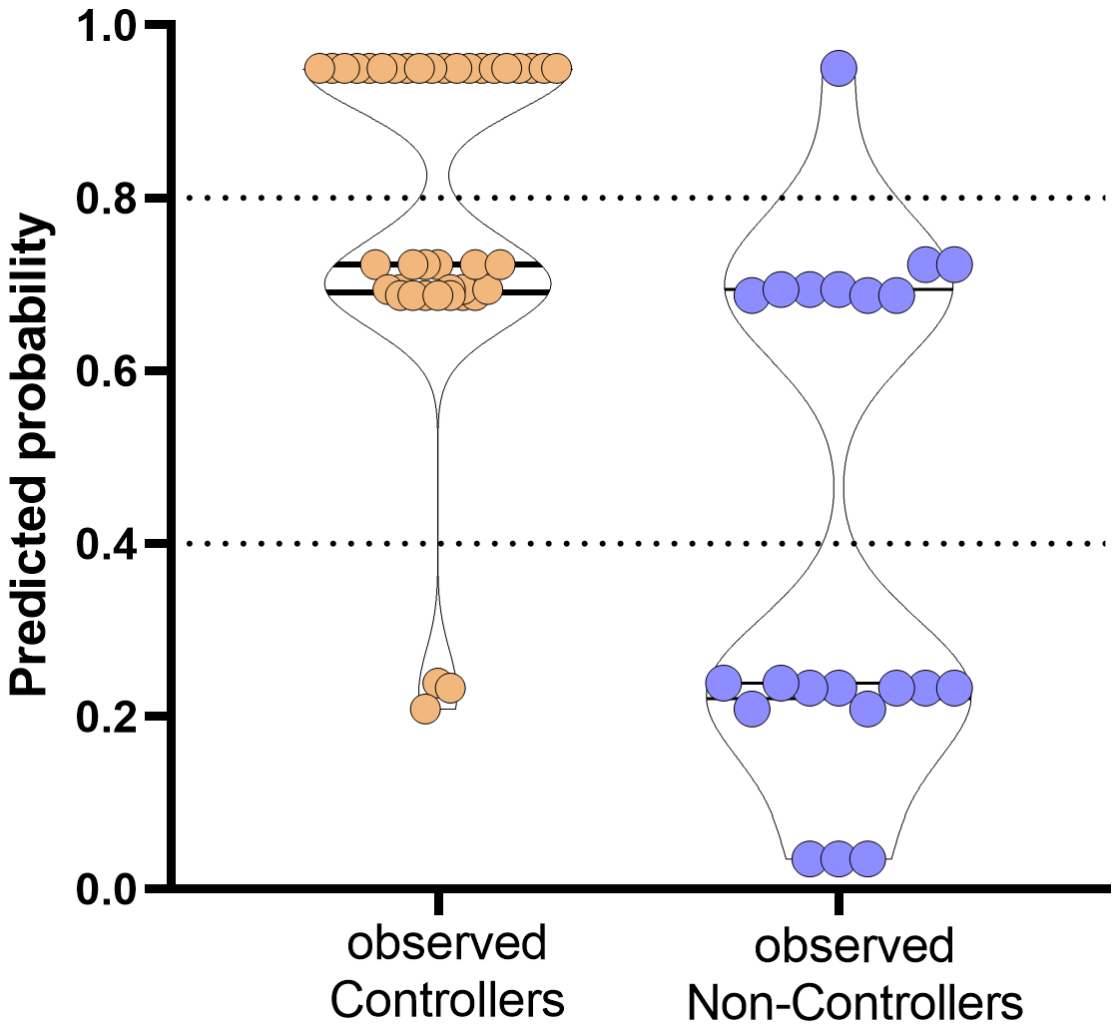
Prediction of probability of the event occurrence in observed Controllers (orange dots) and Non-Controllers (purple dots). The y-axis ranges from 0 (highest probability to be HCMV uncontroller) to 1 (highest probability to be HCMV controller). Dotted horizontal lines have been included for indicating grey zone.


Multivariate logistic analysis for predicting the risk of HCMV Non-Controllers (treated) infection was performed including IE1-specific T-cell response (higher or lower than 60 spots), CD8^+^ T-cell count (higher or lower than 215 cells/µl) and age (higher or lower than 60 years) as variables. Results were given in **Table 2** and **Figure 5**. Mathematical function is the following:

**Table 2:** Multivariate logistic regression.

Odds ratios	Variable	Estimate	95% CI (profile likelihood)	“p value”
β0	Intercept	0,036	0,003082 to 0,2390	0,0022
β1	B: IE1	8,614	2,194 to 42,92	0,0038
β2	C: CD8	7,254	1,841 to 34,98	0,0071
β3	D: age	8,341	1,916 to 46,74	0,0079

Logit[P(Y=1)] = Ln[(P(Y=1)/P(Y=0)] = β0 + β1*B + β2*C + β3*D

Based on the proposed model, the combination of the three variables are able to predict with high probability the rate of controller patients. In detail, percentages of negative and positive predictive power were 80% and 80.85%, respectively. The percentage of correctly classified “Controllers” was 92.7% while the percentage of correctly classified “Non-Controllers” was 57.2%. 

## 4 Discussion

The evaluation of HCMV serostatus in both donor and recipient at time of transplant is considered the most informative approach for the stratification of the risk for HCMV infection after transplant. However, even if HCMV-seropositive recipients are considered to have HCMV-specific immune response, HCMV can reactivate in some patients leading to the risk of HCMV-related complications (30). For this reason, a tool for a better stratification should be introduced, especially for the risk definition among HCMV-seropositive recipients (31).

In this study, we examined the role of lymphocyte count and HCMV-specific T-cell response measured at pre-transplant as potential predictive markers of spontaneous control of HCMV reactivations following kidney transplant. High absolute number of CD8^+^ T cells and sustained IE1-specific T-cell response were independently associated with highest rate of patients with spontaneous resolution of HCMV reactivation (defined as controllers) during the first year after transplant. Moreover, the rate of Controllers was higher in younger subjects. Additionally, even if the difference is not statistically significant, it seems that donor serostatus could have an impact on the occurrence of clinically relevant HCMV reactivations. However, as major limitation of the study, HCMV donor serostatus is unknown for about 30% of the subjects.

Many studies investigated the role of absolute lymphocyte count measured after transplant or at time of treatment in predicting the rate of HCMV infection or recurrent HCMV infection after treatment (10, 32–34). However, the potential role of baseline pre-transplant measurement has been less extensively investigated. In our study, a threshold of IE1-specific T-cell response of 60 net spots/million PBMC was the best cut off for the identification of patients with high probability to control HCMV reactivation spontaneously. Previous studies suggested a possible role of lower IE1-specific T-cell response as risk factor for HCMV reactivation (20, 23, 25, 35). In our study, higher pre-transplant pp65-specific T-cell response was observed in patients with self-resolving HCMV reactivation than in patients with clinically relevant HCMV reactivation, although the difference was not statistically significant. On the contrary, Kim and colleagues reported that pp65-specific T-cell response measured at pre-transplant, but not IE1-specific T-cell response seems to predict the development of HCMV reactivation in HCMV-seropositive patients (36). The reasons for these differences might be related to the type of stimuli used or outcome definition. Further evaluation on this field are necessary. So far, the lack of standardized assays represents a crucial issue for the comparison of results between different clinical settings.

Based on our results, patients with pre-transplant IE1-specific T-cell response above this cut off showed higher probability to develop self-resolving HCMV reactivations. Furthermore, the cumulative incidence of HCMV reactivation events in patients with impaired pre-transplant IE1-specific T-cell response was higher. This means that higher pre-transplant IE1-specific T-cell response could be predictive of sustained immunity in the post-transplant period (15). According to this hypothesis, it was previously observed that patients with positive pre-transplant HCMV-specific T-cell response showed higher HCMV-specific immune response in the post-transplant period. On the contrary, in patients with no pre-transplant HCMV-specific immune response, post-transplant T-cell response specific for HCMV was detectable 3 months after transplant in less than 50% of patients, suggesting a long-term impairment in the control of HCMV infection (21). To date, no universal cut-off of DNAemia have been chosen for starting pre-emptive therapy; for this reason pre-transplant HCMV-specific T-cell response should be evaluated in different transplant setting, in relation to diagnostic and therapeutic protocols. To conclude, in addition to the assessment of HCMV serostatus in patients attending for transplant, pre-transplant IE1-specific T-cell response and CD8^+^ T cell count evaluation should be further investigated for definition of potential algorithm for a better stratification of the risk in HCMV-seropositive recipients and “*ad hoc*” therapeutic strategies, including modulation of immunosuppression therapy.

## Data Availability

The raw data supporting the conclusions of this article will be made available by the authors, without undue reservation.

## Supplementary material

Supplementary Figure 1 Flow-chart representing the enrollment and monitoring of kidney transplant recipients. KTR: kidney transplant recipients; PBMC: peripheral blood mononuclear cells; (*) patients with self-resolving HCMV reactivations or undetectable HCMV DNAemia; (**) patients treated for clinically significant HCMV reactivation
