# Supplementary figures and images for: Pre-transplant IE1-specific T-cell response and CD8+ T-cell count as predictive markers of treated HCMV reactivation in kidney transplant recipients
Source: Front Immunol. 2025 Apr 16;16:1538795. doi: 10.3389/fimmu.2025.1538795 (PMC12040814; doi:10.3389/fimmu.2025.1538795)

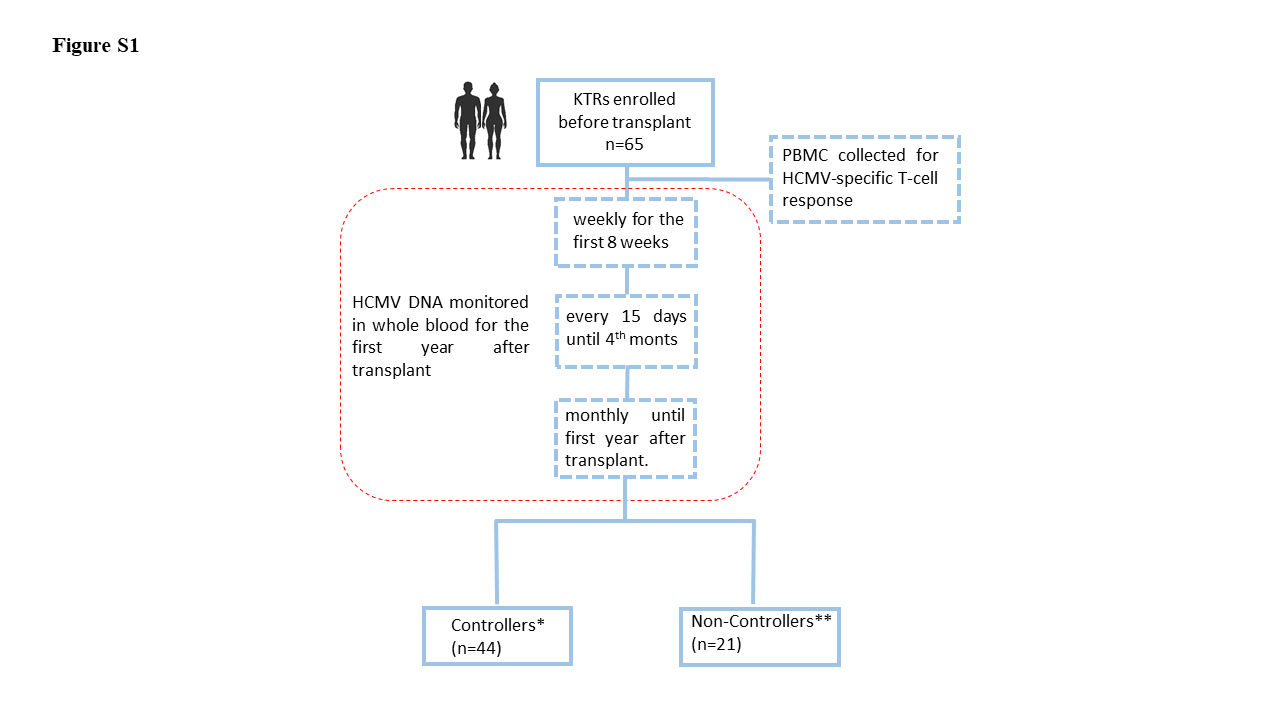

Supplement: Supplementary Figure 1 — Flow-chart representing the enrollment and monitoring of kidney transplant recipients. KTR: kidney transplant recipients; PBMC: peripheral blood mononuclear cells; (*) patients with self-resolving HCMV reactivations or undetectable HCMV DNAemia; (**) patients treated for clinically significant HCMV reactivation [file Image1.tif]
