# Supplementary material for: Pre-transplant IE1-specific T-cell response and CD8+ T-cell count as predictive markers of treated HCMV reactivation in kidney transplant recipients
Source: Front Immunol. 2025 Apr 16;16:1538795. doi: 10.3389/fimmu.2025.1538795 (PMC12040814; doi:10.3389/fimmu.2025.1538795)
Supplement: Supplementary file 2 [file Table1.docx]

**Supplementary Table 1.** ROC curve parameters to predict the spontaneous clearance of HCMV infection

| **Parameters** | **ELISPOT-IE1** | **CD8 T cells/µl** |
| --- | --- | --- |
| **AUC** | 0.68 | 0.67 |
| **CUT-OFF** | 59 | 214 |
| **sensitivity (%)** | 62 | 57 |
| **specificity (%)** | 78 | 77 |
| **PPV (%)** | 76 | 79 |
| **NPV (%)** | 86 | 55 |

**Legend:** AUC, area under curve; PPV, positive predictive value; NPV, negative predictive value
